# Prenatal and early-life determinants of neurodevelopment: A decade of discoveries and new directions in ABCD

**DOI:** 10.1016/j.dcn.2026.101747

**Published:** 2026-05-25

**Authors:** Iris Menu, Arnaud Cachia, Moriah E. Thomason

**Affiliations:** a Université Paris Cité, LaPsyDÉ, CNRS, Paris F-75005, France; b Université Paris Cité, Imaging Biomarkers for Brain Development and Disorders, INSERM, GHU Paris Psychiatrie & Neurosciences, Paris F-75005, France; c Department of Child & Adolescent Psychiatry, NYU Langone Health, New York, NY 10016, United States; d Department of Population Health, NYU Langone Health, New York, NY 10016, United States; e Neuroscience Institute, NYU Langone Health, New York, NY 10016, United States

**Keywords:** Adolescent brain cognitive development (ABCD) Study, Perinatal factors, Prenatal exposure, Neurocognitive development, Brain imaging, Longitudinal cohort

## Abstract

Decades of research on the developmental origins of health and disease highlight how prenatal and perinatal conditions are associated with long-term neurocognitive development. Exposures such as maternal stress, substance use, metabolic disorders, obstetric complications, low birthweight, and preterm birth have been linked to differences in brain, cognition, and mental health. Yet, most prior studies have been limited by small sample sizes, narrow exposure measures, or isolated outcomes. The Adolescent Brain Cognitive Development (ABCD) Study provides an unparalleled opportunity to overcome these limitations with nearly 12,000 children recruited at ages 9–10 across the United States, followed longitudinally with harmonized multimodal MRI, cognitive and behavioral testing, biospecimens, genetic data, and rich environmental measures in a diverse cohort. Retrospective caregiver reports provide key prenatal and perinatal information, which can be prospectively related to neurodevelopmental outcomes across adolescence. This review synthesizes findings from 111 ABCD-based studies published from 2017 to 2026. Results implicate maternal health conditions, substance use, birth outcomes, and cumulative adversity as being associated with variation in brain, cognitive, and behavioral development. Leveraging its size and diversity, ABCD has fostered advanced analytic approaches, such as multimodal integration of imaging and behavioral data, and longitudinal tracking of developmental trajectories, rarely possible elsewhere. While methodological challenges remain, including retrospective reporting and imaging site variability, ABCD offers unique opportunities to clarify pathways of vulnerability and resilience within an observational framework. Insights from this work can inform public health strategies and guide policies to reduce prenatal risks and strengthening early-life environments to optimize developmental outcomes.

## 1 Introduction

Decades of research on the developmental origins of health and disease (DOHaD) underscore the importance of the prenatal period for later neurodevelopment. The fetal programming hypothesis (also known as The Barker Hypothesis) suggests that conditions during embryonic and fetal development can shape long-term physiological and structural characteristics of organs, influencing health trajectories across the lifespan (Kwon and Kim, 2017). This extends to the brain and mental health. Fetal brain development, influenced by factors like maternal stress, inflammation, or environmental exposures, can sow the seeds for heightened susceptibility to psychopathology and neurodevelopmental disorders in offspring (Bale et al., 2010, Buss et al., 2012, Ellison, 2010). For instance, prenatal exposure to stress, undernutrition, or immune challenges can contribute to disorders such as autism spectrum disorder (ASD) and attention deficit hyperactivity disorder (ADHD; Doi et al., 2022). Low birth weight and preterm birth, markers of adverse prenatal environments, are consistently associated with poorer cognitive function and altered brain structure in childhood and beyond (Brydges et al., 2018, Ma et al., 2022). At the same time, these prenatal factors also contribute to variation within the range of typical development: even subtle differences in birthweight or prenatal psychological distress have been linked to brain anatomy and other life outcomes in otherwise healthy populations (De Asis-Cruz et al., 2023, Jha et al., 2019, Silventoinen et al., 2023). To sum up, early-life conditions can cast long shadows: events in the womb can help set developmental trajectories that unfold across childhood and adolescence.

**Fig. 1:**
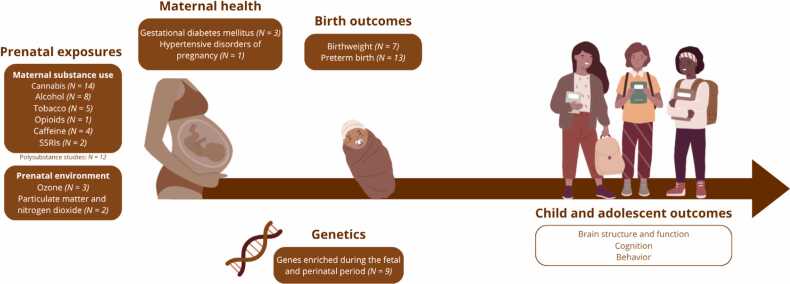
Schematic overview of the ABCD Study design, showing the longitudinal timeline from the prenatal period through adolescence, highlighting the prenatal and perinatal factors captured in ABCD. The number of studies associated with each factor is indicated, though some studies examine related exposures or broader combinations of prenatal and perinatal influences. The factors shown represent a selection of commonly studied exposures in relation to developmental outcomes rather than an exhaustive list of all prenatal and perinatal variables assessed in ABCD. Hypertensive disorders may include pre-eclampsia and related conditions, which are closely linked to placental dysfunction.

Despite this growing body of evidence, most studies to date have relied on relatively small samples, limited exposure assessments, or focused on isolated outcomes. Addressing these limitations requires longitudinal, population-based research with sufficient scale, diversity, and dimensionality to model how multiple prenatal adversities interact with genetic factors and postnatal environments to shape brain and behavioral development. The Adolescent Brain Cognitive Development (ABCD) Study provides an unparalleled resource for addressing this goal. Initiated in the U.S. to chart brain and cognitive development from late childhood into young adulthood (Karcher and Barch, 2021), the study recruited approximately 11,800 children at baseline across 21 sites using stratified probability sampling to reflect national demographics. At baseline, participants are aged 9–10 years (inclusive), and because follow-up assessments occur annually with rolling enrollment, age ranges overlap across study waves. Although ABCD was not originally designed to probe prenatal exposures, its broad scope and multidimensional design make it uniquely suited for this purpose. It collects rich, annual data including MRI scans, cognitive assessments, biospecimens, and detailed environmental and behavioral measures. Retrospective caregiver reports (particularly the Developmental History questionnaire) capture key prenatal and perinatal variables such as birthweight, gestational age, maternal health, and substance exposures during pregnancy (Fig. 1). Importantly, this questionnaire covers a substantially broader range of prenatal and perinatal conditions, including placental and hypertensive disorders (e.g., pre-eclampsia, eclampsia, placental abruption), common gestational complications (e.g., urinary tract infections, severe anemia, severe nausea, unplanned pregnancy), less frequent birth complications (e.g., oxygen deprivation, jaundice, Rh incompatibility), as well as parental age at birth (see ABCD data dictionary for the full list of variables). This design allows researchers to link early-life indices to contemporaneous measures of brain structure and function, cognition, and behavior beginning at ages 9–10, and to track their evolution across adolescence. Importantly, ABCD includes a large and demographically diverse cohort that enables investigations of both population-level patterns and subgroup-specific effects. Subsamples, for example, defined based on developmental comorbidities or socioeconomic strata, allow a more precise study of interrelated factors within select groups. Understanding how associations between biobehavioral and developmental factors shift across subgroups can provide novel foundational understanding and improve study generalizability. Twins are another subgroup that strengthens the overall design, as analyses in twins provides opportunity to disentangle genetic and environmental influences. Thus, ABCD’s large sample, rich phenotyping, and demographically diverse recruitment offer a unique opportunity to test DOHaD-related hypotheses at scale, refining our understanding of how early exposures contribute to individual differences in neurocognitive development.

In this review, we synthesize findings from studies that have used the ABCD dataset to examine prenatal and perinatal influences on brain, cognitive, and behavioral development. We examine associations between maternal health, substance use, and birth outcomes with later neurodevelopmental markers. This review aims to provide an integrative overview of how conditions before birth shape the developing brain and behavior, and to outline how the ABCD study’s rich data can illuminate these developmental origins of neurocognitive outcomes, with particular attention to how such findings can be translated into policy and intervention and to the novel data methods that could be investigated as participants transition into adulthood.

## 2 Methods

To identify relevant studies for this review, we utilized the curated list of publications from the Adolescent Brain Cognitive Development (ABCD) Study, available on the study’s official website (https://abcdstudy.org/research-publications/). This registry includes peer-reviewed articles that meet quality standards established by major indexing databases such as MEDLINE, Web of Science, Scopus, the Directory of Open Access Journals, and the NIH Library. As of April 2026, the database listed a total of 1878 peer-reviewed publications derived from ABCD data. To improve completeness, we additionally conducted a parallel search in MEDLINE/PubMed using the same keyword strategy.

To extract studies focusing on prenatal and perinatal influences on developmental outcomes, we conducted a keyword-based search across all titles and abstracts. The keywords included: *gestational age*, *preterm birth*, *premature birth*, *birth weight*, *placenta, placental complications,* as well as exposure terms constructed using combinations such as *(smoking OR tobacco OR cigarette) AND (maternal OR pregnancy OR prenatal), (alcohol) AND (maternal OR pregnancy OR prenatal), (nutrition OR diet) AND (maternal OR pregnancy OR prenatal), and (environmental exposure OR pollutant) AND (pregnancy OR prenatal OR fetal*), alongside general terms including *fetal, prenatal, and perinatal*. Brain-related terms (*sulcal morphology* and *gyrification*) were also included because these cortical folding features are largely determined during prenatal development and are largely established in the perinatal period, making them sensitive markers of early neurodevelopmental processes (Cachia et al., 2021).

**Fig. 2:**
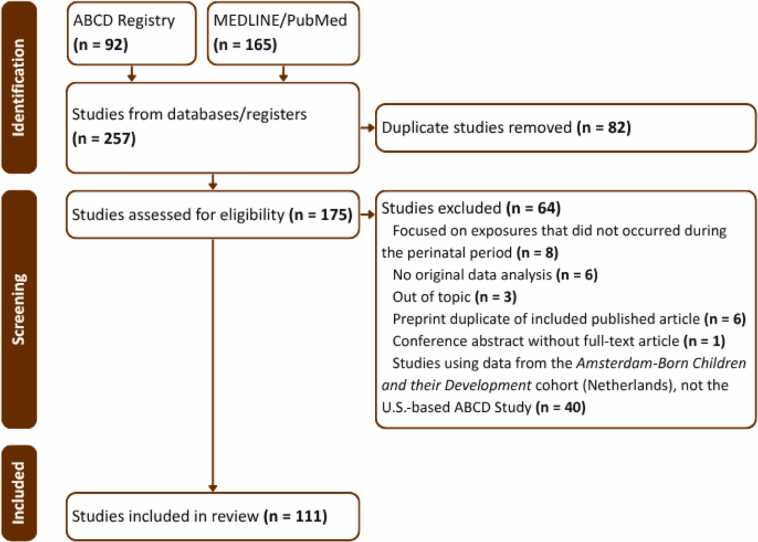
Flowchart of the systematic review search conducted.

This combined search yielded 257 records (MEDLINE/PubMed: n = 165; ABCD registry: n = 92), of which 82 duplicates were removed. The remaining 175 studies were assessed for eligibility based on full-text review. Studies were excluded if they (1) examined exposures that did not occur during the prenatal or perinatal period, (2) did not include original data analyses (e.g., editorials, commentaries), (3) were out of scope, (4) corresponded to preprints superseded by peer-reviewed publications, (5) were conference abstracts without full-text articles, or (6) used data from a different cohort (e.g., the Amsterdam-Born Children and their Development cohort rather than the U.S.-based ABCD Study). Importantly, no restrictions were applied based on outcome domains (e.g., behavioral or neuroimaging measures), allowing inclusion of all studies assessing developmental outcomes following perinatal exposures. Following this screening process, a final sample of 111 studies was retained for inclusion in the review (Fig. 2).

## 3 Synthesis of findings

In this section, we synthesize evidence from 111 studies using the ABCD dataset published between 2017 and 2026 on prenatal and early-life factors, including substance and pollutant exposures, birthweight and gestational age, maternal health conditions, genetic influences, and their interactions, that shape brain, cognitive, and behavioral development.

### 3.1 Prenatal substance exposure

Prenatal exposure to psychoactive substances (including cannabis, tobacco, alcohol, opioids and combinations thereof) has long been implicated in adverse neurodevelopmental outcomes (Akison et al., 2024, Karatayev et al., 2024, Sheffield et al., 2024). In ABCD (Table 1), retrospective maternal reports collected via the Developmental History Questionnaire assess prenatal substance use before and after pregnancy recognition, enabling analyses of timing-specific associations.

**Table 1:** Summary of ABCD-based studies examining prenatal substance exposures. Note: Due to the longitudinal and rolling enrollment design of the ABCD Study, age ranges overlap across waves; therefore, all results are organized by study time point (e.g., baseline, 1-year follow-up) rather than exact age at assessment.

**Study**	**N**	**Early factor(s) studied**	**Main outcome(s) assessed**	**Key findings**
*Cannabis exposure*				
Acosta-Rodriguez et al. (2025b)	1085 (baseline), 418 exposed, 667 matched controls	Prenatal cannabis exposure	Structural MRI Diffusion MRI	Reduced white matter integrity and cortical surface area in frontal and parietal regions; associations with attention-related problems
Amir et al. (2025)	11,368 (baseline), 7928 (2-year follow-up), 2982 (4-year follow-up), 652 exposed	Prenatal cannabis exposure	fMRI (Monetary Incentive Delay task) Psychotic-like experiences	Reduced activation linked to psychotic-like experiences, suggesting altered dopaminergic development
Baranger et al. (2022)	10,624 (baseline), 10,094 (1-year follow-up), 9373 (2-year follow-up), 599 exposed	Prenatal cannabis exposure	Psychopathology (Child Behavior Checklist, Psychotic-like experiences)	Persistent elevations in psychopathology into early adolescence
Baranger et al. (2024)	9322–10186 (baseline and 2-year follow-up), 568 exposed	Prenatal cannabis exposure	Neuroimaging (structural MRI, diffusion MRI and resting-state fMRI) Psychopathology (Child Behavior Checklist, Psychotic-like experiences)	Localized differences in gray and white matter; altered resting-state connectivity; partial mediation of attention problems
Cioffredi et al. (2022)	896 (baseline), 224 exposed, 672 matched controls	Prenatal cannabis exposure after knowledge of pregnancy	Behavior (Child Behavior Checklist, Brief Problem Monitor – Teacher) Neurocognition (NIH Toolbox) fMRI (Stop Signal, EN-Back and Monetary Incentive Delay tasks)	Greater attention and externalizing problems despite no detectable task-based fMRI activation differences
Evanski et al. (2024)	11,530 (baseline), 697 exposed	Prenatal cannabis exposure	Diffusion MRI	Reduced fractional anisotropy in fornix
Faraj et al. (2023)	10,719 (baseline), 564 exposed	Prenatal cannabis exposure	Resting-state fMRI	Lower coupling between salience and ventral attention networks; mediates associations with psychotic-like symptoms
Fine et al. (2019)	4361 (baseline), 201 exposed	Prenatal cannabis exposure	Psychosis proneness (Prodromal Questionnaire)	Exposure after pregnancy awareness associated with increased childhood psychosis proneness; no effect for exposure before awareness
Fu et al. (2026)	10,836 (baseline), 754 exposed	Prenatal cannabis exposure	Resting-state fMRI Psychopathology (Child Behavior Checklist) Neurocognition (NIH Toolbox)	Altered functional network connectivity, higher psychopathology, and subtle cognitive differences
Hiraoka et al. (2023)	11,530 (baseline and 2-year follow-up), 697 exposed	Prenatal cannabis exposure	Structural MRI Neurocognition (NIH Toolbox, Little Man Test)	Associations with visuo-perceptual processing and intracranial volume intensify over time
Paul et al. (2021)	11,489 (baseline), 890 exposed	Prenatal cannabis exposure	Structural MRI Psychopathology (Child Behavior Checklist, Psychotic-like experiences) Neurocognition (NIH Toolbox)	Increased risk of internalizing and externalizing symptoms; differences persisted after controlling for confounders
Spechler et al. (2023)	9825 (baseline and 1-year follow-up), 605 exposed	Prenatal cannabis exposure	Sleep duration Psychopathology (Child Behavior Checklist)	Protective effect of longer sleep duration reduced among PCE-exposed children; associated with more behavioral problems
Vishnubhotla et al. (2024)	178 (baseline), 88 exposed, 90 matched controls	Prenatal cannabis exposure	Neuroimaging (structural MRI, diffusion MRI and resting-state fMRI) Psychopathology (Child Behavior Checklist)	PCE moderates associations between brain network organization and behavior; no large-scale morphometric differences
Winiger and Hewitt (2020)	11,875 (baseline), 695 exposed	Prenatal cannabis exposure	Sleep quality	Lower overall sleep quality compared to non-exposed peers
*Alcohol Exposure*				
Agarwal et al. (2025)	6830	Prenatal alcohol exposure	Behavior (BIS/BAS scale, UPPS-P Impulsive Behavior Scale, Child Behavior Checklist) Neuroimaging (structural and fMRI - Monetary Incentive Delay task)	Associated within broader risk profiles linked to altered reward processing and increased impulsivity/behavioral problems
Devine et al. (2025)	10,336 (3-year follow-up), 2582 exposed	Prenatal alcohol exposure	Sleep quality (Sleep Disturbance Scale for Children)	Poorer adolescent sleep outcomes, with non-linear associations even at low exposure levels
Gerlikhman and Sarkar (2024)	4996 (baseline and 1-year follow-up), 494 exposed	Prenatal alcohol exposure Polygenic risk scores for mental health problems	Psychopathology (Child Behavior Checklist)	Polygenic risk scores moderated the association between PAE and child psychopathology
Lees, Mewton, Jacobus et al. (2020)	9719 (baseline), 2518 exposed	Prenatal alcohol exposure	Psychopathology (Child Behavior Checklist, Kiddie Schedule for Affective Disorders and Schizophrenia) Neurocognition (NIH Toolbox)	Greater internalizing and externalizing symptoms, attentional difficulties, reduced cortical thickness
Lees, Mewton, Stapinski et al. (2020)	10,119 (baseline), 2675 exposed	Prenatal alcohol exposure	Early alcohol sipping (iSay Sip Inventory)	Increased odds of early alcohol sipping, suggesting influence on later alcohol-related behaviors
Long and Lebel (2022)	270 (baseline), 135 exposed, 135 matched controls	Prenatal alcohol exposure after knowledge of pregnancy	Structural MRI Psychopathology (Child Behavior Checklist)	Structural differences in white matter and cortical volume even with low exposure
Long and Lebel (2026)	216 (baseline – 4-year follow-up), 108 exposed, 108 matched controls	Low-level prenatal alcohol exposure	Structural MRI Psychopathology (Child Behavior Checklist)	Persistent behavioral problems and longitudinal brain differences compared
Maxwell et al. (2026)	9253 (baseline)	Prenatal alcohol exposure	Early alcohol sipping (iSay Sip Inventory)	Causal risk factor for early alcohol sipping, with gender-specific developmental pathways
*Tobacco Exposure*				
Gonzalez et al. (2023)	8803 (baseline), 1099 exposed	Prenatal tobacco exposure	Physical health (body mass index) Structural MRI Neurocognition (NIH Toolbox)	Differences in child physical health and neurodevelopment
Kochvar et al. (2025)	11,448 (baseline), 1607 exposed	Prenatal tobacco exposure	Structural MRI	Reduced cortical surface area/thickness and altered sulcal depth; region-specific cortical maturation differences
Puga, Doucet, et al. (2024)	11,448 (baseline), 1607 exposed	Prenatal tobacco exposure	Structural MRI	Reduced gray-white matter contrast and smaller subcortical volumes, longitudinally stable
Puga, Dai, et al. (2024)	11,448 (baseline), 1607 exposed	Prenatal tobacco exposure	Structural MRI Neurocognition (NIH Toolbox)	Reduced cortical morphometry and lower language/crystallized cognition performance
Rodriguez Rivera et al. (2023)	11,609 (baseline), 620 exposed	Prenatal tobacco exposure	Structural MRI Neurocognition (NIH Toolbox)	Widespread cortical/subcortical reductions; poorer cognitive performance; partial brain-mediated associations (with sex differences)
*Opioid exposure*				
Hartwell et al. (2020)	11,530 (baseline), 150 exposed	Prenatal opioid exposure	Structural MRI	Reduced volume in precentral gyrus
*Caffeine exposure*				
Agarwal et al. (2022)	5534 (baseline), 4412 exposed	Prenatal caffeine exposure	Total sugar intake (Block Kids Food Screener Questionnaire) Physical health (body mass index) fMRI (Monetary Incentive Delay task)	Higher caffeine use linked to increased sugar intake, higher BMI; altered reward sensitivity
Christensen et al. (2021)	9157 (baseline), 4135 exposed	Prenatal caffeine exposure	Diffusion MRI Neurocognition (NIH Toolbox) Psychopathology (Child Behavior Checklist)	White matter alterations and increased psychopathology, with limited cognitive associations
Modi et al. (2025)	10,873 (baseline), 6560 exposed	Prenatal caffeine exposure	Physical health (birth outcomes, puberty status, body mass index) Psychopathology (Child Behavior Checklist, Psychotic-like experiences) Sleep quality	Elevated BMI but no behavioral difference (except sleep) after multiple comparisons correction
Zhang et al. (2022)	9978 (baseline), 5808 exposed	Prenatal caffeine exposure	Structural MRI Physical health (body mass index) Psychopathology (Child Behavior Checklist) Sleep quality Neurocognition (NIH Toolbox, Little Man Test)	Alterations in brain structure and function; increased externalizing behaviors
*SSRIs exposure*				
Moreau et al. (2023)	5420–7528 (baseline), 235 exposed	Prenatal SSRI exposure	Structural MRI Psychopathology (Child Behavior Checklist) Maternal Depression (Adult Self-Report Depressive Problems DSM-5-Oriented Scale)	Small increase in depressive symptoms; differences in cortical thickness and surface area in localized regions
Zanni et al. (2025)	3973 (2-year follow-up), 97 exposed	Prenatal SSRI exposure	fMRI (EN-Back task) Psychopathology (Child Behavior Checklist)	Greater amygdala activation; elevated anxiety and depression symptoms
*Polysubstance*				
Gu et al. (2024)	9838 (baseline), 5880 exposed to prenatal caffeine, 2524 exposed to PAE, 1300 exposed to PTE, 547 exposed to PCE, 2587 exposed to polysubstance	Prenatal alcohol, marijuana, tobacco and caffeine exposure	Structural MRI resting-state fMRI Psychopathology (Child Behavior Checklist) Neurocognition (NIH Toolbox) Impulsivity (Urgency, premeditation, perseverance, sensation seeking, positive urgency, and impulsive behavior scale) Inhibition (Behavioral Inhibition System and Behavioral Activation System scales)	Associations with adverse health and cognitive outcomes reduced when accounting for environmental and genetic factors
Hartwell et al. (2024)	11,755 (baseline), 2892 exposed to PAE, 1604 exposed to PTE, 149 exposed to POE	Prenatal opioid, alcohol and tobacco exposure	Structural MRI	Smaller parahippocampal volume
Kohler et al. (2022)	11,045 (baseline), 1474 exposed	Prenatal tobacco, alcohol and marijuana exposure	Impulsivity (delay-discounting)	No clear difference in delay-discounting
Lepow et al. (2024)	6146 (baseline), 394 exposed	Prenatal tobacco, alcohol and marijuana exposure	fMRI (EN-Back task) Childhood trauma (Kiddie Schedule for Affective Disorders and Schizophrenia)	Altered responses to fearful vs. happy faces; some differences emerge only with co-occurring trauma
R. Li et al. (2025)	7881 (baseline to 4-year follow-up)	Prenatal tobacco, alcohol, caffeine and cannabis exposure	Physical health (tri-ponderal mass index)	Increased risk of rising adiposity
Q. Li et al. (2026)	7777 (baseline), 848 exposed to PTE, 2084 exposed to PAE, 336 exposed to PCE	Prenatal tobacco, alcohol and cannabis exposure	Psychopathology (Child Behavior Checklist)	Substance-specific and timing-dependent associations with psychopathology, particularly in relation to maternal pregnancy awareness
Marshall et al. (2025)	9792 (baseline), 772 exposed to PTE, 1516 exposed to PAE	Prenatal tobacco and alcohol exposure	Structural MRI Psychopathology (Child Behavior Checklist) Impulsivity (Urgency, premeditation, perseverance, sensation seeking, positive urgency, and impulsive behavior scale) Sleep quality	Persistent differences in cortical development linked to higher externalizing behaviors and sleep problems
Nadler et al. (2025)	9792 (baseline), 290 exposed to PCE and PTE, 225 exposed to PCE only, 966 exposed to PTE only	Prenatal tobacco and cannabis exposure	Psychopathology (Child Behavior Checklist)	Greater problems compared to exposure to either substance alone or none
Orendain et al. (2023)	11,566 (baseline)	Early life adversity exposure including prenatal substance exposure	Psychopathology (Child Behavior Checklist)	Higher externalizing problems
Paige et al. (2026)	11,029 (baseline), 2772 exposed to PAE, 217 exposed to PCE, 10,967 (2-year follow-up), 4754 (4-year follow-up)	Prenatal alcohol and cannabis exposure	Neurocognition (NIH Toolbox)	Little evidence for adverse associations on cognitive development after covariate adjustment; small positive associations for PAE, no combined exposure associations
Vishnubhotla et al. (2026)	6674 (baseline),262 exposed to PTE, 180 exposed to PAE, 105 exposed to PCE, 7 exposed to prenatal cocaine and 9 exposed to prenatal opioid exposure	Prenatal tobacco, alcohol, cannabis, cocaine and opioid exposure	Psychopathology (Child Behavior Checklist) resting-state fMRI	PTE and PCE exposure were associated with worse behavioral outcomes; PTE linked to altered large-scale network connectivity related to behavioral symptoms
Xia and Vieira (2025)	7887 (baseline), 2041 exposed	Prenatal tobacco and alcohol exposure, Neighborhood environmental exposures (including NO2, PM, Ozone)	Structural MRI	High residential deprivation associated with smaller hippocampal volumes only in children without PTE

#### 3.1.1 Cannabis

Rates of prenatal cannabis exposure (PCE) have increased alongside changes in legalization and social norms, prompting greater scientific interest in its potential neurodevelopmental correlates. In our review, 11 studies specifically examined PCE and most focused on brain structure, function, and brain-behavior associations. Diffusion MRI studies have reported microstructural differences in frontal and parietal white-matter tracts, as well as regional variations in cortical morphology, among PCE-exposed youth (Acosta-Rodriguez et al., 2025b, Baranger et al., 2024). For instance, Evanski and colleagues (2024) reported small but reliable reductions in fractional anisotropy of the fornix, a key frontolimbic tract involved in emotion and memory, highlighting PCE’s subtle associations with white matter integrity during childhood. Baranger and colleagues (2024) also reported region-specific alterations in gray and white matter within the frontal and parietal cortices, their associated tracts, and striatal resting-state connectivity, with some diffusion metrics partially mediating associations between PCE and attention-related problems.

Behavioral findings suggest that timing of exposure may be critical. Fine and colleagues (2019) reported that PCE after, but not before, maternal awareness of pregnancy was associated with higher psychosis proneness in middle childhood, whereas earlier exposure showed no significant associations after adjustment for confounders. Paul and colleagues (2021) similarly found links between PCE and higher childhood psychopathology risk (including both internalizing and externalizing problems), particularly when exposure occurred after maternal awareness of pregnancy. These associations persisted after controlling for confounding factors such as maternal education, socioeconomic status, and other prenatal exposures, whereas exposure only prior to pregnancy awareness showed no significant associations. Of note, these models adjusted for familial, pregnancy (including PAE and PTE), and child-level covariates (including child substance use), without restricting the sample to cannabis-naïve children. Baranger and colleagues (2022) extended these findings longitudinally, showing that associations between PCE and psychopathology persisted into early adolescence, reinforcing the developmental stability of these differences.

Functional and network-level analyses further reveal altered neural dynamics. Fu and colleagues (2026) demonstrated that PCE is associated with altered large-scale functional network connectivity, alongside higher psychopathology and subtle cognitive differences, with exposure-related connectivity patterns overlapping with both behavioral and cognitive phenotypes. Reduced ventral striatal activation during reward anticipation has been observed in youth with PCE, which was associated with greater psychotic-like experiences, a pattern that may reflect altered development of dopaminergic circuitry (Amir et al., 2025). Lower functional coupling between the salience and ventral attention networks, particularly when exposure occurred before maternal awareness of pregnancy, mediated associations with psychotic-like symptoms, though these differences disappeared after covariate adjustment (Faraj et al., 2023). Additionally, graph-theoretical analyses indicate that PCE may moderate associations between brain network organization and behavior, even in the absence of large-scale morphometric differences (Vishnubhotla et al., 2024). However, PCE has been linked to greater attention and externalizing problems, even in the absence of detectable task-based fMRI activation differences (Cioffredi et al., 2022). Crucially, longitudinal studies hint that these neural perturbations may not be static. Over time, PCE-related associations with visuo-perceptual processing and intracranial volume appear to intensify, suggesting a progressive divergence from typical developmental trajectories (Hiraoka et al., 2023).

Sleep has also emerged as an important domain of PCE investigation. One study found that children with PCE reported lower overall sleep quality at ages 9–10 compared to non-exposed peers (Winiger and Hewitt, 2020), suggesting possible disruptions in sleep regulation. Another study reported that the protective effect of longer sleep duration, typically associated with fewer internalizing and externalizing symptoms, was significantly reduced among children with PCE (Spechler et al., 2023).

Taken together, these studies suggest that PCE is associated with subtle, distributed differences in brain structure, connectivity, and function (rather than overt structural abnormalities) which may underlie later cognitive and behavioral outcomes, underscoring the need for continued longitudinal research to clarify these developmental pathways.

#### 3.1.2 Alcohol

In parallel, research has also explored the potential neurodevelopmental and behavioral correlates of prenatal alcohol exposure (PAE), including at low to moderate levels, a focus reflected in the four studies identified in our review. Lees, Mewton, Jacobus, and colleagues (2020) reported that PAE was associated with a broad range of psychological, behavioral, and neurodevelopmental outcomes in substance-naive children. Agarwal and colleagues (2025) further identified PAE as one of the key factors stratifying children into higher-risk groups for maladaptive behaviors, with these profiles showing altered reward-related activation, increased impulsivity, and greater behavioral problems at follow-up. Specifically, PAE was linked to greater internalizing and externalizing symptoms, attentional difficulties, and differences in brain structure, including greater cerebral and regional volume and greater regional surface area. A related study (Lees, Mewton, Stapinski, et al., 2020) found that even low levels of PAE were associated with increased odds of early alcohol sipping by age 9–10, suggesting that early *in utero* exposure may influence later alcohol-related behaviors, potentially through alterations in cognitive control or reward sensitivity pathways. Similarly, Maxwell and colleagues (2026) used causal discovery methods to show that PAE and broader family-level drinking norms were linked to early alcohol sipping, highlighting the relevance of both prenatal exposure and familial context in the early emergence of alcohol-related behavior.

Expanding on these associations, Long and Lebel (2022) examined brain alterations in children with low-level PAE and found structural differences in white matter structure, despite the relatively low exposure levels and a demographically matched control group. A longitudinal follow-up by the same team showed that low-level PAE was associated with persistently higher behavioral problems and greater intracranial volume across early adolescence, indicating that these differences may endure over time (Long and Lebel, 2026). Devine and colleagues (2025) found that any PAE was associated with poorer adolescent sleep outcomes, with evidence of non-linear dose associations at low exposure levels. Together, these findings challenge the notion of a strict dose-response threshold and suggest that subtle brain and behavioral changes may occur even with limited prenatal exposure. In a more recent study, Gerlikhman and Sarkar (2024) explored how genetic susceptibility interacts with PAE to influence mental health outcomes. Their results indicated that polygenic risk scores for mental health disorders moderated the association between PAE and child psychopathology, highlighting the complex interplay between genetic and environmental influences on developmental trajectories.

Together, these studies underscore that PAE, even at low to moderate levels, may be associated with measurable differences in brain structure, behavioral functioning, and early alcohol-related behaviors. Furthermore, individual differences in genetic vulnerability may shape how PAE manifests, pointing to the importance of considering both biological and environmental factors in understanding developmental risk.

#### 3.1.3 Tobacco

Tobacco has been also studied, with five studies focusing exclusively on prenatal tobacco exposure (PTE). Gonzalez and colleagues (2023) examined the associations between PTE and both physical health and neurodevelopmental outcomes, considering intersecting sociodemographic and prenatal factors. Their findings suggest that PTE relates to differences in child physical health and neurodevelopment, with early postnatal health potentially mediating these associations, including shorter breastfeeding duration, lower birthweight, and being small for gestational age, which were also linked to increased risk of childhood obesity and poorer cognitive and brain outcomes, particularly with longer duration of exposure. At the neural level, Puga, Doucet, and colleagues (2024) reported longitudinal associations between PTE and reduced gray/white matter contrast as well as smaller subcortical volumes, including the caudate nucleus, with differences persisting from ages 9–12. Kochvar and colleagues (2025) found that PTE was associated with lower intracranial volume, reduced cortical surface area and thickness, and altered sulcal depth in specific regions, including parahippocampal and cingulate areas, suggesting broad but regionally specific differences in cortical maturation trajectories. Extending these findings to cognitive outcomes, Puga, Dai, and colleagues (2024) showed that PTE was associated with reduced cortical morphometry in frontal, parietal, and medial temporal regions, alongside lower performance in language-related and crystallized cognition domains. Similarly, Rodriguez Rivera and colleagues (2023) reported widespread reductions in cortical surface area and subcortical volumes, as well as poorer performance across multiple cognitive domains, with mediation analyses suggesting that structural brain differences partially account for PTE-related cognitive differences, along with some sex-specific differences in selected regions. Overall, these studies converge in suggesting that PTE is associated with distributed alterations in brain structure and neurocognitive development rather than focal abnormalities.

#### 3.1.4 Opioids

Our review identified one study examining the neurodevelopmental relates to prenatal opioid exposure (POE) within the ABCD cohort. Hartwell and colleagues (2020) conducted a cross-sectional analysis comparing children with and without reported POE, controlling for sociodemographic confounders. The authors found that POE was associated with reduced volume in the precentral gyrus, a brain region implicated in motor function. Although this structural difference did not extend to broader cortical measures, the findings suggest a potential association between POE and region-specific brain morphology.

#### 3.1.5 Caffeine

Prenatal caffeine exposure is widespread, yet its long-term neurodevelopmental and behavioral implications remain under investigation. In our review, we identified three studies examining associations between prenatal caffeine exposure and child outcomes using data from the ABCD Study. Zhang and colleagues (2020) first reported that prenatal caffeine exposure was associated with alterations in brain structure and function in children aged 9–11, including lower frontal thickness and greater occipital and posterior cingulate thickness. These structural differences were accompanied by higher externalizing behaviors, particularly in males, as well as elevated BMI for exposures above 200 mg per day. Associations with sleep, internalizing problems, and pubertal development were attenuated after adjusting for covariates, highlighting the influence of parental and household factors. Christensen and colleagues (2021) has also linked gestational caffeine exposure to alterations in white matter microstructure, particularly in fronto-occipital and corticospinal tracts, with associations to increased psychopathology but minimal differences on cognitive performance, suggesting that caffeine-related differences may extend to distributed brain connectivity. Agarwal and colleagues (2022) further examined prenatal caffeine exposure, dietary behaviors, and reward processing. Higher prenatal caffeine use, particularly daily or weekly, was linked to increased sugar intake and higher BMI in adolescents. Neuroimaging showed greater insular thickness and reduced middle frontal cortex activation during reward anticipation. Mediation analyses indicated that altered reward sensitivity may partly explain the association with BMI. These results suggest that prenatal caffeine exposure can shape neural circuits involved in food preference and reward, potentially increasing obesity risk. Most recently, Modi and colleagues (2025) examined longitudinal associations between prenatal caffeine exposure and both behavioral and physical health outcomes as children transition into adolescence. Consistent with earlier work, they found that prenatal caffeine exposure was positively associated with elevated BMI but, after correcting for multiple comparisons, it was not associated with child behavioral outcomes, except for sleep problems in children exposed to two or more cups daily.

#### 3.1.6 SSRIs

We identified two studies investigating the associations of prenatal exposure with selective serotonin reuptake inhibitors (SSRIs) on child neurodevelopment and behavior. SSRIs are commonly prescribed during pregnancy, yet their long-term impact on the developing brain remains an area of active investigation. Using data from the ABCD Study, Moreau and colleagues (2023) examined whether prenatal SSRI exposure was associated with depressive symptoms and brain morphology in middle childhood. They found that exposed children showed a small increase in depressive symptoms, especially for exposure before maternal awareness, though this was no longer significant after accounting for recent maternal depression. MRI analyses revealed thicker left lateral occipital cortex and larger left superior parietal surface area in exposed children, but these structural differences were not linked to depressive symptoms, and no associations were found for other prenatal medications.

In a more recent translational study combining human and animal models, Zanni and colleagues (2025) investigated how perinatal SSRI exposure affects fear-related brain circuit activation and behavior. In mice, early-life SSRI exposure resulted in heightened innate defense responses to predator odors and increased activation in amygdala-centered fear circuits. Paralleling these findings in humans, adolescents from the ABCD study with prenatal SSRI exposure showed greater activation of the amygdala and related limbic regions when processing fearful facial expressions, alongside elevated symptoms of anxiety and depression compared to non-exposed peers. These cross-species findings suggest that SSRI exposure may influence the development of neural circuits involved in fear and emotion processing, with observable differences emerging in adolescence.

Overall, these studies point to potential associations between prenatal SSRI exposure and subtle differences in brain structure and function, particularly in emotion-related regions, but with modest behavioral differences in childhood. Moreover, they demonstrate the value of the ABCD Study not only as a resource for examining long-term, population-level outcomes of prenatal exposures, but also as a translational platform that can be aligned with mechanistic animal models. Finally, from a broader perspective, patterns of antidepressant use during pregnancy may reflect underlying social determinants: non-native English speakers were more likely to discontinue treatment after pregnancy recognition, highlighting potential language-related disparities in both exposure and maternal mental health care (Dupuis et al., 2024).

#### 3.1.7 Polysubstance and contextual moderation

This section focuses on studies examining prenatal exposure to multiple substances within the same analytic framework (see Table 1 for substances investigated per study). Importantly, most included studies do not assess true simultaneous polysubstance use (i.e., concurrent exposure to multiple substances within individuals), but rather model multiple substance exposures (e.g., tobacco, alcohol, cannabis, opioids) within the same study design either as separate predictors or in combination, allowing for comparison of substance-specific and shared associations.

Exposure to multiple substances appears to influence developmental trajectories in intertwined ways. For example, Marshall and colleagues (2025) examined PTE and PAE as separate predictors and found that PTE, but not PAE, was associated with subtle, region-specific differences in cortical development between ages 9 and 13, including faster thinning in frontal regions. These cortical changes were linked to higher externalizing behaviors and sleep problems, suggesting that tobacco exposure may modestly alter brain maturation trajectories rather than causing widespread disruptions. Hartwell and colleagues (2024) examined multiple prenatal substance exposures (opioids, tobacco, and cannabis) within the same analytic framework and reported that prenatal tobacco exposure is linked to smaller parahippocampal volume, an alteration with plausible downstream effects on memory, motor development, and behavior. Converging evidence from functional imaging further indicates that PTE and PCE are associated with alterations in large-scale network connectivity, particularly within default mode and attention networks linked to behavioral symptoms (Vishnubhotla et al., 2026). Xia and Vieira (2025) showed that neighborhood environment interacts with prenatal alcohol and tobacco exposures to shape hippocampal and parahippocampal volumes in late childhood: high residential deprivation was associated with smaller hippocampal volume only among children without prenatal tobacco exposure, suggesting that prenatal factors can buffer or amplify postnatal environmental risks. Prenatal exposures also predict later physical health trajectories. R. Li and colleagues (2025) found that multi-substance prenatal exposure increases the risk of rising adiposity from pre- to early adolescence, highlighting how these early exposures may contribute to later physical health challenges like obesity, with tobacco and caffeine showing particularly strong, dose-dependent associations.

Behaviorally, co-exposure to prenatal cannabis and tobacco has been to greater externalizing problems in middle childhood compared to exposure to either substance alone or none, with higher tobacco exposure potentially amplifying cannabis associations with both externalizing and internalizing behaviors, indicating additive or interactive associations on emotional and behavioral regulation (Nadler et al., 2025). Building on this, Q. Li and colleagues (2026) reported substance-specific vulnerability windows, with pre-awareness alcohol exposure and post-awareness cannabis exposure showing the clearest links to childhood psychopathology, whereas PTE showed more modest differences across timings. At the same time, evidence for combined low-level exposures remains mixed, with Paige and colleagues (2026) finding minimal adverse associations of low PAE and PCE on cognitive trajectories after accounting for sociodemographic factors. However, in an ABCD subsample, Kohler and colleagues (2022) reported no difference in delay-discounting (a potential risk marker for early substance-use initiation) between 9- to 10-year-olds with and without prenatal exposure, suggesting that some behavioral differences may be subtle or emerge later. A very small increase was observed when maternal substance use occurred after knowledge of pregnancy, although this effect was minimal.

Importantly, the picture is further complicated by genetic and familial risk factors. Using a data-driven analysis of early life adversity, Orendain and colleagues (2023) identified prenatal substance exposure as one of six core adversity domains, alongside parental psychopathology, neighborhood threat, scarcity, and household dysfunction. Gu and colleagues (2024) integrated these contexts and found that while prenatal exposures to substances are associated with adverse health and cognitive outcomes, many of these associations are substantially reduced when accounting for broader environmental and genetic factors. Notably, the interplay between prenatal drug exposure and childhood experiences emerges as critical. Indeed, Lepow and colleagues (2024) found that prenatal drug exposure altered neural responses to fearful versus happy faces, with some differences present regardless of childhood trauma and others emerging only when exposure co-occurred with trauma, the latter linking to internalizing and externalizing behaviors (see Table 1 for the specific substances included in each study). This highlights that prenatal substance exposure does not operate in isolation but within a dynamic matrix of risks and protections shaping developmental trajectories.

In summary, the ABCD study has provided critical insights into prenatal substance exposure by integrating extensive neuroimaging, behavioral, genetic, and environmental data to examine how PSE interacts with postnatal contexts to influence developmental outcomes. While these findings advance understanding of prenatal influences on brain and behavior, they should be interpreted in light of potential confounding from shared familial environment, parental psychopathology, genetic liability, and correlated postnatal exposures that are not consistently accounted for across studies. This “health in context” approach reveals that the associations with prenatal exposures, such as alcohol, tobacco, caffeine, cannabis, opioids, and SSRIs, cannot be fully understood in isolation and are best understood as embedded within broader developmental systems involving genetic predisposition and environmental factors like neighborhood deprivation, childhood trauma, and familial risk (Gu et al., 2024, Lepow et al., 2024, Marshall et al., 2025). Results from ABCD studies highlight the importance of examining modifiable environmental and social factors that may buffer or exacerbate PSE-related risks, such as neighborhood quality, early intervention programs, and family support. Understanding risk as complex and multifactorial is essential for the advancement of precision medicine frameworks tailored to the intricate biopsychosocial contexts that shape outcomes after prenatal exposure.

### 3.2 Prenatal pollution

Prenatal exposure to environmental pollutants, particularly airborne contaminants such as particulate matter (PM) and nitrogen dioxide (NO₂), has been proposed as a potential disruptor of early brain development. These exposures can affect fetal development through inflammatory, oxidative stress, and endocrine pathways, potentially leading to long-term alterations in neural structure and function (e.g., Volk et al., 2021). In the ABCD Study, air pollution exposure is estimated using geocoded residential history, allowing for large-scale investigations of environmental risk across developmental timepoints, including during gestation.

Szwed and colleagues (2025) examined whether prenatal and childhood exposure to PM and NO₂ were associated with myelin-sensitive MRI markers, using the T1w/T2w ratio as a proxy for cortical myelin content. They found a marginal negative association between prenatal NO₂ exposure and cortical myelin signal, though this effect appeared confounded by scanner-related variability and regional sociodemographic factors, underscoring the methodological complexity of assessing environmental exposure associations across multisite datasets. A separate longitudinal analysis by Kong and colleagues (2025) further found that higher prenatal ozone exposure interacted with adverse school and neighborhood contexts at ages 9–10 to predict reduced limbic system volume, poorer cognitive functioning, and elevated psychotic-like experiences at ages 11–13. Notably, neither prenatal pollution nor psychosocial adversity alone showed strong associations with these outcomes. Still, their interaction suggests a cumulative or synergistic effect on vulnerable neural circuits, particularly those related to emotion regulation and salience processing.

Damme and colleagues (2026) extended this work by examining cumulative exposure to fine PM across prenatal and late childhood periods in relation to hippocampal structure and cognition. They found that higher PM exposure was associated with smaller hippocampal volumes and poorer working memory performance, with the strongest differences observed among youth exposed during both developmental windows. These findings suggest that air pollution may exert additive or cumulative influences on neural development, particularly in regions such as the hippocampus that exhibit prolonged plasticity. On the other hand, Cotter and colleagues (2024) found that pollution exposure, but particularly during childhood rather than prenatal periods, was associated with white matter microstructure in early adolescence, with patterns differing by sex. Extending this work by examining potential protective factors, Cotter and colleagues (2025) found that children with longer and more efficient sleep showed attenuated associations between pollution exposure (prenatal ozone and childhood nitrogen dioxide exposure) and altered white matter microstructure organization, suggesting that sleep may function as a protective factor against environmental neurotoxicity.

Together, these studies provide preliminary but important insights into how prenatal exposure to air pollutants may influence child brain development, either independently or in combination with social environmental risks. Recent findings further suggest that these associations may not be limited to a single sensitive period but instead accumulate across prenatal and later developmental stages, with variability depending on timing and individual characteristics, including sex (Cotter et al., 2024). Emerging evidence also highlights the role of moderating factors such as sleep, which may buffer the association with environmental neurotoxicity, underscoring the interplay between risk and resilience processes (Cotter et al., 2025). Importantly, in ABCD, pollution exposure is estimated from geocoded residential addresses using ensemble-based models that integrate satellite-derived and land-use information, and most studies model exposures within a specific developmental window (e.g., prenatal) rather than lifetime cumulative exposure; therefore, reported prenatal associations should be interpreted as window-specific estimates rather than fully isolated gestational effects. Although the observed associations were modest and in one case confounded by technical limitations or measurement variability, the findings support the relevance of considering prenatal environmental exposures as part of a broader risk framework for understanding variability in neurodevelopmental trajectories. Moreover, the integration of geospatial environmental data with high-resolution neuroimaging in ABCD offers a scalable platform for further research in this area.

### 3.3 Preterm birth and low birthweight

Preterm birth, typically defined as delivery before 37 weeks of gestation, and low birthweight (LBW; <2500 g), including very low birthweight (VLBW; <1500 g), are well-established indicators of prenatal adversity with long-term consequences for brain development, cognitive performance, and behavior (e.g., Brydges et al., 2018; de Kieviet et al., 2012; Houdt et al., 2019). In the ABCD Study (Table 2), these variables are captured retrospectively through caregiver responses to the Developmental History Questionnaire.

**Table 2:** Summary of ABCD-based studies on preterm birth and birthweight. Note: Due to the longitudinal and rolling enrollment design of the ABCD Study, age ranges overlap across waves; therefore, all results are organized by study time point (e.g., baseline, 1-year follow-up) rather than exact age at assessment.

**Study**	**N**	**Early factor(s) studied**	**Main outcome(s) assessed**	**Key findings**
Acosta-Rodriguez et al. (2025a)	3323 (baseline), 613 preterm, 2710 full-term	Moderate to late preterm birth	Neurocognition (NIH Toolbox) Neuroimaging (structural and diffusion MRI, resting-state fMRI)	Frontal-lobe structural and functional alterations mediate the link between gestational age and executive function in moderate-to-late preterm children.
Dahl et al. (2024)	7760 (baseline)	Birthweight	Neurocognition (NIH Toolbox) Behavior (Child Behavior Checklist) Genetics Structural MRI	Cortical thickness correlated with birthweight independent of genetic similarity, supporting a direct effect of somatic growth on brain morphology.
Dooley et al. (2022)	9706 (baseline)	Birthweight	Behavior (Child Behavior Checklist)	Lower birthweight linked to more attention problems, especially in boys; weaker links with other psychopathological domains.
Dooley et al. (2023)	8835 (ABCD baseline) + 7724 children from *Growing Up in Ireland* study	Restricted fetal growth	Behavior (Child Behavior Checklist)	Familial and prenatal factors partly explained fetal growth-ADHD association with differences across cohorts.
Ji et al. (2023)	3571 (baseline), 1706 preterm, 1865 matched-controls	Preterm birth	Neurocognition (NIH Toolbox) Behavior (Child Behavior Checklist) Structural MRI	Preterm children showed greater deviations in gray and white matter volume compared to term peers, indicating lasting neuroanatomical differences.
Ji et al. (2024)	3571 (baseline), 1706 preterm, 1865 matched-controls	Birthweight	Adrenarcheal hormones Neurocognition (NIH Toolbox) Structural MRI	Lower birthweight associated to altered hormones and lower cortical and regional volume, contributing to poorer memory performance.
Ji et al. (2025)	3571 (baseline), 1706 preterm, 1865 matched-controls	Very low birthweight, preterm birth	Adrenarcheal hormones Neurocognition (NIH Toolbox) Behavior (Child Behavior Checklist) Neuroimaging (structural and diffusion MRI)	VLBW preterm children showed higher cortical thickness, reduced cortical area, reduced subcortical and tract volumes, linked to lower cognition, altered pubertal timing, and higher psychopathology risk.
H. Li et al. (2025)	612 (baseline), 306 preterm, 306 matched-controls	Preterm birth	Neurocognition (NIH Toolbox) Diffusion MRI	Differences in white matter microstructure in frontal-limbic tracts; altered associations between structural connectivity and attention/processing speed performance.
Ma et al. (2022)	11,878 (baseline), 5685 (2-year follow-up) + 618,070 adolescents from the Danish national register	Gestational age	Neurocognition (NIH Toolbox, Little Man Task, Rey Auditory Verbal Learning Test, Matrix Reasoning Task) Structural MRI	Lower GA linked to smaller cortical volume and reduced academic scores, independent of postnatal environment.
Menu, Duffy et al. (2025)	3508 (baseline and 2-year follow-up), 1754 preterm, 1754 matched-controls	Preterm birth	“Cool” vs. “Hot” executive functions (Flanker, Emotional Stroop, Delay Discounting tasks) Structural MRI	Persistent deficits in “cool” EF, partial catch-up in “hot” EF, indicating differential maturational trajectories; different cognitive-brain maturation relationships in preterm children.
Menu, Ji et al. (2025)	1891 (baseline and 2-year follow-up)	Preterm birth	Neurocognition (NIH Toolbox) Behavior (Child Behavior Checklist) Neuroimaging (structural MRI and resting-state fMRI) School grades	Latent profiles based on neurocognition revealed subgroups (persistently low, heterogeneous, above average); linked to brain structure and connectivity, behavior, and academic achievement.
Nath et al. (2023)	9667 (baseline)	Gestational age, birthweight	Structural MRI	Preterm birth linked to cortical thinning (temporoparietal, DLPFC) and thickening (mPFC, occipital); associations partly mediated by birthweight.
Nivins, Padilla, Kvanta, and Ådén (2025)	5946 (baseline)	Gestational age	Neurocognition (NIH Toolbox, Little Man Task, Rey Auditory Verbal Learning Test)	Each week below term linked to lower cognitive scores, even after genetic/prenatal covariates.
Nivins, Padilla, Kvanta, Mårtensson, et al. (2025)	7281 (baseline), 222 preterm, 7056 full-term	Preterm birth	Neurocognition (NIH Toolbox) Structural MRI	Prematurity-specific neural alterations underpin low cognitive performance in preterm youth, varying by degree of prematurity.
Nolte et al. (2024)	8349 (baseline), 1615 preterm, 6734 full-term	Preterm birth	Vocabulary (*Picture Vocabulary* from the NIH Toolbox) Structural MRI	Additive associations of prematurity + Socioeconomic status on cortical thickness, predicting poorer vocabulary.
Retzler et al. (2023)	11,381 (baseline), 1544 preterm	Preterm birth	Neurocognition (NIH Toolbox) Behavior (Child Behavior Checklist) Diffusion MRI	No evidence for a unique “preterm behavioral phenotype”; prematurity linked to general risk, not syndrome-specific patterns.
Senger-Carpenter et al. (2024)	5645 girls (baseline), 1039 preterm	Preterm birth	Early puberty (Pubertal Development Scale)	Premature birth is associated with early pubertal timing alongside BMI and multiple social and familial adversity indicators.
Taylor et al. (2025)	2899 (baseline and 1-year follow-up)	Preterm birth	Inhibition (Flanker Task) Diffusion MRI	Preterm birth associated with reduced FA and higher MD in uncinate fasciculus/cingulum; predicted weaker inhibition, especially under socioeconomic disadvantage.
Walhovd et al. (2024)	5718 from ABCD, Lifespan Changes in Brain and Cognition and UK Biobank cohorts	Birthweight	Structural MRI	Birthweight positively predicted brain volume and surface area across life.
Zhou et al. (2024)	5922 (baseline), 4949 appropriate / 331 small / 642 large for gestational age	Birthweight (small/large for gestational age)	Neurocognition (NIH Toolbox) Behavior (Child Behavior Checklist) Structural MRI	U-shaped effect: both small and large for gestational age linked to differences in brain volume and area vs. appropriate for gestational age peers but no cognitive or behavioral differences.

Several studies using ABCD data have documented widespread differences in cortical and subcortical brain structure associated with earlier gestational age and lower birthweight. For instance, Nath and colleagues (2023) identified cortical thinning in temporoparietal and dorsolateral prefrontal cortices in children born preterm, with concurrent thickening observed in medial prefrontal and occipital areas. These regional alterations were partially mediated by birthweight, suggesting that growth restriction may underlie some of the observed morphological differences. Similarly, Ji and colleagues (2023) reported that preterm children showed greater deviations in both gray and white matter volume relative to full-term peers, supporting the notion that early perinatal insults continue to shape brain development into late childhood. The same team further showed that preterm children with VLBW exhibited a distinct profile of higher cortical thickness, reduced cortical area, and reduced subcortical volumes across widespread regions, alongside attenuated white-matter tract volumes (Ji et al., 2025). These alterations were linked to lower cognitive functioning, altered pubertal timing, and elevated psychopathology risk, highlighting distinctive developmental deviations in this subgroup. Acosta-Rodriguez and colleagues (2025a) further demonstrated that moderate-to-late preterm birth (of note, representing the largest proportion of preterm births) is also associated with widespread neurodevelopmental alterations, particularly in frontal regions supporting executive functions. Reduced white-matter integrity and functional connectivity in these networks mediated small but significant decrements in cognitive performance, emphasizing the sensitivity of frontal systems to even moderate reductions in gestational age. Another study demonstrated that preterm children with low cognitive performance exhibited distinct regional and network-level differences, including thinner inferior temporal and fusiform cortices, larger amygdala and smaller hippocampal volumes, and atypical structural covariance between parietal and sensorimotor hubs (Nivins et al., 2025). These neural alterations were modestly associated with ADHD and ASD symptoms, highlighting potential compensatory mechanisms and the need for prematurity-tailored interventions. Dahl and colleagues (2024) demonstrated that individual differences in cortical thickness are systematically associated with anthropometrics, including birthweight, even after accounting for genetic similarity. This reinforces the conclusion that birthweight exerts a direct and measurable influence on brain morphology, underscoring its role as a key biological pathway through which preterm birth and growth restriction shape neurodevelopment. Extending these observations into adolescence and adulthood, Walhovd and colleagues (2024) drew on data from the ABCD study for childhood and adolescence and from additional cohorts spanning adulthood (Lifespan Changes in Brain and Cognition and the older adult UK Biobank) to show that birthweight is positively associated with cortical surface area and brain volume not only in youth but also across adulthood. These findings suggest that early somatic growth may lay the foundation for long-term brain reserve.

White-matter connectivity is also affected by prematurity, with implications for executive functioning. Executive functions, which include working memory, inhibitory control, and cognitive flexibility, are crucial for adaptive, goal-directed behavior (Diamond, 2013). Taylor and colleagues (2025) demonstrated that preterm birth is associated with reduced fractional anisotropy and increased mean diffusivity in tracts implicated in inhibitory control, including the uncinate fasciculus and cingulum bundle. These structural differences predicted poorer performance on response inhibition tasks and were amplified in the context of socioeconomic disadvantage, illustrating how biological vulnerability may be exacerbated by environmental risk. Interestingly, H. Li and colleagues (2025) showed that even medically non-complex preterm children exhibit reduced white matter integrity in frontal-limbic tracts, particularly affecting attention and processing speed networks. Consistent with this and with broader literature on executive functioning (e.g., Houdt et al., 2019), Menu, Duffy, and colleagues (2025) found that deficits in "cool" executive functions (i.e., emotionally neutral tasks) persisted from late childhood through early adolescence among preterm children. In contrast, "hot" executive functions (i.e., emotionally salient decision-making tasks) showed evidence of developmental catch-up, highlighting differential maturational trajectories in affective versus cognitive control systems.

Beyond executive functioning, several studies have examined the associations of gestational age and birthweight with broader cognitive outcomes. Nivins and colleagues (2025) found that cognitive performance, assessed via the NIH Toolbox, showed a graded association with gestational age. Specifically, each additional week of prematurity below term was linked to lower cognitive scores, even after controlling for genetic and prenatal factors. Ma and colleagues (2022) similarly observed that lower gestational age predicted smaller cortical volume and lower scores on academic achievement tests. Notably, these differences were independent of postnatal environmental variables, suggesting enduring associations of gestational timing on neurocognitive development. Zhou and colleagues (2025) examined how deviations in birthweight relate to brain development in early adolescence, finding that both small- and large-for-gestational-age children showed altered brain volume and area compared to appropriate-for-gestational-age peers, despite no differences in cognitive or behavioral performance. Complementing this, Ji and colleagues (2024) demonstrated that lower birthweight was linked to altered adrenarcheal hormone profiles and reduced cortical and regional brain volumes, which in turn contributed to poorer memory performance. Together, these findings underscore the complexity of birthweight as both a marker of prenatal adversity and a biological influence on neurodevelopmental processes.

At the same time, not all preterm-born youth display the same cognitive challenges. Using latent profile modeling, Menu, Ji, and colleagues (2025) identified distinct subgroups of preterm youth based on baseline performance across seven NIH Toolbox tasks: while about 40% showed persistently low neurocognitive functioning, around 20% performed above average. These cognitive profiles were associated with differences in brain structure, functional connectivity, behavior, and academic achievement, and remained evident two years later. Nolte and colleagues (2024) demonstrated that prematurity and socioeconomic disadvantage exert additive associations on cortical thickness, which in turn predicted receptive vocabulary. Together, these findings highlight that prematurity confers probabilistic rather than deterministic risk, with outcomes shaped by both individual neurobiological variability and broader environmental conditions.

In addition to cognitive outcomes, behavioral problems have been a major focus of research on prematurity and birthweight. Dooley and colleagues (2022) identified robust associations between lower birthweight and increased attention problems, particularly in boys, while other domains of psychopathology showed weaker associations. The same team extended this work by comparing ABCD and Growing Up in Ireland cohorts and found that more than one-quarter of the fetal growth-ADHD association was attributable to familial confounds, with prenatal contributions differing by cohort: pregnancy complications explained a larger proportion in ABCD, whereas maternal substance use explained more in Growing Up in Ireland (Dooley et al., 2023). However, Retzler and colleagues (2023), using latent class analysis of the Child Behavior Checklist, found no evidence of a distinct “*preterm behavioral phenotype*” (i.e., difficulties with emotions, attention, and peer relationships behavioral and emotional difficulties typically observed in ADHD, ASD, and anxiety disorders; Fitzallen et al., 2020). Instead, their findings suggest that preterm birth is associated with broad, nonspecific behavioral risk rather than a unique pattern of psychopathology. Beyond behavioral outcomes, Senger-Carpenter and colleagues (2024) found that premature birth contributed to earlier pubertal timing within a broader model of layered adversity, suggesting that gestational age interacts with socioeconomic, familial, and individual-level stressors in shaping pubertal developmental trajectories.

### 3.4 Maternal metabolic and hypertensive disorders

Gestational diabetes mellitus (GDM), characterized by glucose intolerance first identified during pregnancy, and hypertensive disorders of pregnancy (HDP), including gestational hypertension and preeclampsia, are increasingly recognized as prenatal factors with implications for offspring neurodevelopment. Notably, hypertensive disorders, particularly pre-eclampsia, are closely linked to placental dysfunction, and growing research highlights the role of placental programming mechanisms in shaping fetal brain development (Kokori et al., 2024, Lewis et al., 2025). In the ABCD Study, exposure to these conditions is also captured retrospectively through caregiver responses in the Developmental History Questionnaire.

Ahmed and colleagues (2023) reported that children exposed to maternal diabetes exhibited reduced cortical thickness across several regions, most notably in the occipital lobes, postcentral gyrus, and superior parietal cortex. These alterations, though modest in magnitude, were statistically significant after adjusting for sociodemographic and perinatal covariates and were accompanied by lower cognitive performance, particularly in processing speed. In a separate study, Luo and colleagues (2023) found that GDM-exposed children had higher BMI and waist-related measures, with these associations partially mediated by alterations in brain volume within the medial orbitofrontal cortex, anterior cingulate, and precuneus, regions involved in reward, regulation, and interoception, suggesting links between prenatal metabolic exposure, brain development, and later health. Complementing this work, Ahmed and colleagues (2024) investigated HDP exposure and found no direct differences in cortical thickness or neurocognitive functioning between exposed and unexposed children. However, birthweight and BMI emerged as key mediators of the relationship between HDP and cognitive outcomes, underscoring how vascular complications of pregnancy may exert indirect developmental effects through fetal growth and somatic pathways. Finally, extending this literature longitudinally, Hsu and colleagues (2026) found that youth exposed to GDM showed greater increases in BMI before puberty and faster cortical thinning after pubertal onset compared to unexposed peers, suggesting that prenatal metabolic exposure may shape developmental trajectories of both somatic growth and brain structure across adolescence.

It should be noted that the effect sizes across these studies were generally small; however, they provide converging evidence that maternal metabolic and hypertensive disorders during pregnancy are associated with structural brain differences and related behavioral or physiological outcomes in preadolescence. As with other prenatal exposures in ABCD, the retrospective nature of the data and lack of specificity regarding timing, severity, and treatment limit interpretability. Nevertheless, these findings highlight the importance of considering multiple maternal health factors during pregnancy in shaping early brain and cognitive trajectories. Future longitudinal analyses, particularly as the cohort progresses through puberty and into adolescence, will be critical for determining the persistence and clinical relevance of these associations.

### 3.5 Genetic studies

In addition to environmental and perinatal factors, genetic variation contributes substantially to early neurodevelopment. Studies using ABCD data highlight these prenatal genetic influences and their links to brain structure and behavior. Tissink and colleagues (2024) first demonstrate extensive genetic overlap across structural, functional, and diffusion MRI measures, with cross-modal genetic factors showing strong prenatal expression patterns, suggesting that shared genetic architecture contributes to coordinated brain development across modalities. Building on this, Patel and colleagues (2022) show that interregional cortical differences associated with psychiatric and behavioral outcomes are linked to genes expressed in prenatal cell types such as radial glia and intermediate progenitors, further supporting a prenatal transcriptional basis for neurodevelopmental vulnerability. Similarly, Cheng and colleagues (2022) identify shared genetic loci between schizophrenia and subcortical brain volumes, many of which show peak expression during prenatal development. Bhatt and colleagues (2025) report that genetic variants associated with corpus callosum morphometry are enriched for prenatal cellular organization and growth processes, and show overlap with cortical features and neuropsychiatric risk, further supporting a shared genetic architecture rooted in early brain development. Extending these findings to behavioral outcomes, Hughes and colleagues (2023) report that a cross-disorder polygenic score indexing neurodevelopmental psychiatric symptoms (including ADHD, autism, depression, and Tourette-related symptoms) is associated with brain structure and function, with implicated genes showing enriched prenatal expression in the fetal cerebellum. Finally, Eising and colleagues (2022) show that genetic associations with reading and language abilities are enriched in regulatory regions active in the fetal brain, highlighting the role of early neurodevelopmental processes in shaping later cognitive outcomes.

These “enrichment” findings are derived by combining genome wide association studies (GWAS) or polygenic data with external maps of gene expression or regulatory activity. Trait-associated single nucleotide polymorphisms (SNPs) are tested for overrepresentation in fetal enhancers or aggregated to genes highly expressed in fetal tissue, while imaging-genetics approaches relate polygenic scores or spatial brain patterns to these expression maps. Importantly, these methods do not measure fetal gene expression in participants, but indicate that the genetic architecture of a trait points to early neurodevelopmental processes. Studies like the ones from Cheng and colleagues (2022) and Hughes and colleagues (2023) further link shared or polygenic variation to brain structure and psychiatric outcomes, highlighting how genes interact with developmental timing and environmental exposures. In a preprint, Forsyth and colleagues (2025) show that deletions affecting genes regulating fetal brain development are associated with lower cognitive functioning and alterations in brain structure, further supporting the role of early gene regulatory processes in shaping neurodevelopmental outcomes. Adding to this, Choi and colleagues (2022) integrated genomic and environmental data (comprising 133 variables capturing family, peer, school, neighborhood, and life event factors) to identify distinct profiles of genetic and exposomic influence on internalizing and externalizing symptoms, highlighting gene-environment interactions that emerge early in development during childhood, with only minimal additional contribution from perinatal exposures.

Importantly, genetic factors do not necessarily exert their influence exclusively before birth; many genes continue to be expressed postnatally, and their effects can interact dynamically with environmental exposures throughout development. Epigenetic mechanisms, including DNA methylation and histone modifications, provide a flexible interface by which early experiences can modify gene expression across the lifespan, further shaping neurodevelopmental trajectories (Peña, 2025). Together, these findings underscore a critical window during fetal development in which genetic programming in regions such as the cerebellum and thalamus may shape later cognition and psychopathology, while also highlighting that postnatal experiences and ongoing gene expression continue to influence developmental outcomes. Considering this dynamic interplay emphasizes the importance of both prenatal and postnatal timing when interpreting genetic influences and identifying opportunities for early intervention.

### 3.6 Interaction and cumulative effects of multiple early-life factors

Finally, research using the ABCD Study highlights that the neurodevelopmental impact of early-life exposures may also arise from a confluence of interrelated prenatal and perinatal risk factors rather than isolated influences. Multivariate and integrative analytic approaches have proven essential for capturing this complexity and its enduring associations with brain, cognition, and behavior. Lindseth and colleagues (2025) applied a data-driven dimensionality reduction technique to distill pregnancy- and birth-related factors into four latent domains (maternal pregnancy complications, maternal substance use, low birthweight/prematurity, and newborn complications) and related these dimensions to global and regional cortical volumetric measures, including patterns of surface area, thickness, and cortical curvature derived from linked independent component analysis of child MRI data. They found that while maternal complications and low birthweight/prematurity were linked to smaller global cortical surface area, newborn complications were associated with regionally specific cortical thickness and surface area patterns, suggesting overlapping and regionally distinct correlates of prenatal and perinatal adversity. Of note, although curvature contributed only minimally in this study, it is of interest given its relationship to sulcal morphology and early cortical development. Building on this framework, Larson and colleagues (2025) derived six dimensions of perinatal insults (substance exposure, obstetric complications, birth complications, postnatal challenges, parental age, and medical needs) from 31 perinatal variables in 11,417 youth aged 9–14 years. The same team further identified five robust dimensions of perinatal adversity, specifically, obstetric complications, birth complications, prenatal substance exposure, parental circumstances, and environmental toxins, and found that certain perinatal dimensions (e.g., parental circumstances) merged with conceptually related childhood adversities such as socioeconomic status, whereas other dimensions (e.g., obstetric complications) remained distinct from childhood adversities such as neglect, emphasizing the importance of type and timing in early-life risk (Larson and Moussa-Tooks, 2025). Other ABCD studies similarly show that early-life risks cluster across domains: for instance, Qiu and Liu (2023) identified integrated pathways linking environmental exposures, brain structure, and psychopathology, while Modabbernia and colleagues (2021) reported multiple population-level modes of covariation spanning perinatal history, cognition, and mental health, supporting the value of integrative, multivariate frameworks. Similarly, H. Zhang and colleagues (2020) identified clusters of parental, socioeconomic, and perinatal factors, showing that broader social and familial contexts (parental psychopathology and socioeconomic status) are more strongly associated with child cognition and behavior than isolated prenatal exposures, further reinforcing the importance of considering co-occurring influences. On the other hand, Dooley and colleagues (2024) identified 17 pre- and perinatal variables that robustly predicted ADHD symptoms, but the overall model explained only about 8% of variance and showed marked heterogeneity across sociodemographic subgroups. This suggests that while perinatal factors contribute to developmental risk, their predictive utility is limited and strongly moderated by broader contextual factors. These multivariate studies also highlight an important interpretational issue: several associations are attenuated after adjustment for familial and socioeconomic context, suggesting that shared family environment and correlated parental factors may explain part of the observed effect. Yet not all studies directly model genetic liability or family clustering, limiting separation of prenatal exposure effects from inherited or shared environmental confounding.

Converging evidence nevertheless highlights the additive effects of multiple prenatal and perinatal adversities. Roffman and colleagues (2021) showed that aggregated prenatal burden, including maternal substance use, pregnancy complications, and psychosocial stressors, was associated with elevated child psychopathology. Staines and colleagues (2024) further found that cumulative prenatal and perinatal adversity increased the risk of psychotic experiences, with persistent symptoms showing the strongest associations. Wiggs and colleagues (2025) reported that diverse prenatal and perinatal contributors to cognitive disengagement syndrome (a neurodevelopmental profile characterized by excessive daydreaming, mental fog, and slowed cognitive processing) likely act through shared developmental bottlenecks such as restricted oxygen or nutrient supply. Complementing this, Adise and colleagues (2024) demonstrated that perinatal complications, including low birthweight, prematurity, and obstetric complications, along with concurrent childhood sleep and behavioral problems, predicted physical health problems at ages 9–10, including sleep disturbances, highlighting clustering of perinatal and current health risks as potential targets for intervention. Of note, no single perinatal factor emerged as uniquely dominant, although low birthweight and prematurity-related indicators showed relatively stronger associations in sensitivity analyses. Shchetinina and colleagues (2026) further showed that combinations of prenatal alcohol exposure and low birthweight were associated with externalizing behaviors in a sex-specific manner. Zhi and colleagues (2026) demonstrated that increasing burden of adverse prenatal exposures was associated with dose-dependent increases in psychopathology and accelerated cortical thinning across adolescence, reinforcing the idea that multiple early risks interact rather than operate independently.

Other studies have delineated neurodevelopmental pathways through which perinatal factors shape later outcomes. Zhi and colleagues (2024) showed that healthy perinatal development predicted better cognitive abilities, while sleep problems, family conflict, and adverse school environments increased risk for mental health problems, with perinatal exposures among the most influential predictors of cognition. On another hand, Carozza and colleagues (2025) found that multiple adversities, including prenatal risks, interpersonal adversity, and socioeconomic disadvantage, were linked to lower fractional anisotropy, which in turn predicted deficits in receptive language and math, suggesting that interregional connectivity mediates behavioral outcomes. Wu and colleagues (2025) similarly reported that perinatal health factors, including birthweight and gestational age, shaped the developmental trajectory of structural brain asymmetry via corpus callosum maturation, which subsequently predicted crystallized intelligence in early adolescence. Zhou and colleagues (2025) demonstrated that twin status was associated with lower cognitive (NIH Toolbox) but better behavioral outcomes (Child Behavior Checklist), alongside smaller cortical volume and area, associations that persisted after accounting for prematurity, birthweight, sibling presence, and zygosity. Yen and colleagues (2026) also showed that prenatal factors such as maternal smoking and gestational age are associated with alterations in hypothalamic volume from birth through adolescence. Finally, Karcher and colleagues (2022) and Kochunov and colleagues (2023) show that both genetic liability and early-life risk factors (including pre/perinatal and environmental exposures) are associated with brain patterns resembling those observed in psychopathology, particularly schizophrenia, suggesting shared neurodevelopmental pathways linking early risk, brain organization, and later mental health outcomes. These ABCD studies underscore the idea that complex perinatal configurations can yield both risk and resilience.

Several ABCD studies highlight the moderating role of environmental and social factors in buffering or amplifying perinatal risk. Gonzalez and colleagues (2020) identified latent factors linking perinatal well-being, psychosocial support, and economic resources to higher cortical surface area and better cognitive performance, with differences strongest in lower-income households, underscoring how positive early ecologies can mitigate adversity. Alnæs and colleagues (2020) examined how patterns of brain structure covary with cognitive, behavioral, and environmental profiles in the population, testing the hypothesis that specific brain-behavior configurations co-occur at the population level. They identified three distinct modes of brain-behavior covariation, one of which reflected perinatal complications (e.g., preterm birth, eclampsia, toxemia, shorter breastfeeding) associated with cortical alterations and lower cognitive scores, illustrating the multidimensional interplay of perinatal, social, and environmental influences. Extending this cross-contextual perspective, Dooley and colleagues (2024) compared cohorts and found that while fetal growth restriction consistently predicted higher ADHD symptom burden, the relative contributions of specific prenatal factors differed across studies: pregnancy complications were more influential in ABCD, whereas maternal substance use explained more variance in the Growing Up in Ireland cohort. Ren and colleagues (2024) and Baker and colleagues (2023) demonstrated that associations between perinatal factors, here maternal age, and child outcomes are strongly shaped by socioeconomic and family context, while Wang and colleagues (2025) showed that environmental factors, including family context, account for a substantial portion of the cognitive-mental health link, further emphasizing the embeddedness of prenatal exposures within broader social systems.

Collectively, these studies show that early-life risk factors often interact and accumulate, converging on shared neurodevelopmental pathways. Findings from the ABCD Study support multifactorial models that capture the diversity of prenatal and perinatal exposures and their combined effects on brain structure, connectivity, cognition, mental health, and physical health. Many associations reaffirm long-established findings (e.g., links between low birthweight and cognitive performance, or between prenatal substance exposure and later psychopathology) thereby strengthening confidence in prior evidence through replication at scale. However, the scope and design of ABCD also allow researchers to move beyond replication: to refine causal interpretations by integrating multimodal data (e.g., mediation of cognitive outcomes through white matter connectivity; Carozza et al., 2025), and to test generalizability across diverse sociodemographic subgroups (e.g., differences in risk pathways for ADHD between U.S. and Irish cohorts; Dooley et al., 2023). More recent work using machine learning and exposomic approaches (Niklason et al., 2025, Pries et al., 2022, Siddique and Lee, 2024) further underscores the value of integrating genetic, environmental, and developmental data to capture small, cumulative effects across multiple domains (respectively, alcohol sipping in childhood, psychosis, and adolescent psychopathology). In this way, the ABCD Study not only consolidates what is known about the developmental consequences of early-life risk but also enhances interpretability and external validity, offering a framework for identifying small, cumulative effects that shape trajectories of both vulnerability and resilience across the population.

## 4 Methodological considerations

Several methodological considerations warrant attention when interpreting findings from the ABCD study. Here, we consider ABCD studies that focus on prenatal and perinatal exposures. A central limitation of these is the reliance on retrospective parental reports. Families were enrolled when children were 9–10 years old. Thus, retrospective recall was subject to potential recall error and misclassification (see also Roffman et al., 2021). Factors such as obstetric complications are recalled with reasonable accuracy (Jaspers et al., 2010), but others, particularly maternal substance use during pregnancy, are frequently underreported, introducing exposure misclassification that may attenuate associations toward the null and, in some cases, may even distort effect estimates. This limitation is especially important for single caregiver-reported items (e.g., maternal alcohol or tobacco use during pregnancy), which may be more vulnerable to recall bias than prospectively collected exposure measures (e.g., obstetric records of gestational age or birth weight). As a result, associations based on these exposures should be interpreted cautiously, particularly when the underlying exposure is stigmatized or likely to be systematically underreported. For example, 60.6% of women in a population-based study reported alcohol use between conception and recognition of pregnancy (McCormack et al., 2017), a rate substantially higher than caregiver-reported ones in ABCD (e.g., 19.3–27.6% in Roffman et al., 2021). Neuroimaging assessments also have limitations. Despite harmonized protocols (Hagler et al., 2019), scanner manufacturer, model, and periodic upgrades can introduce systematic biases, which may affect estimates of cortical thickness and white matter properties (Szwed et al., 2025). Across ABCD neuroimaging studies, these site-related associations are most commonly handled by including scanner or acquisition site as covariates in statistical models (e.g., Ji et al., 2023; Paul et al., 2021). Some studies (e.g., Menu et al., 2025, Yen et al., 2026) additionally applies formal harmonization approaches such as ComBat or longitudinal extensions like longCombat (Beer et al., 2020), which aim to reduce between-site variability prior to analysis. However, the extent of adjustment is not fully consistent across the literature, and not all studies report harmonization procedures or conduct explicit sensitivity analyses to assess robustness across scanners or sites. Harmonization methods mitigate but do not eliminate these effects, and results should therefore be interpreted in light of both residual scanner-related variability and differences in site-adjustment strategies across studies. In addition, analytic approaches to multiple comparison correction vary across ABCD imaging studies. Many studies employ standard procedures such as false discovery rate (FDR) (e.g., Baranger et al., 2022) or Bonferroni (e.g., Menu, Ji, et al., 2025) correction, particularly in whole-brain analyses, whereas others use more limited correction strategies or focus on predefined regions of interest. This heterogeneity should be considered when comparing findings across studies, as differences in correction strategies may influence both the detection and apparent robustness of reported associations.

Sample collection and representativeness pose additional challenges. Extreme prematurity (<28 weeks of gestation), children with cerebral palsy, prior brain hemorrhage, and those in special needs classrooms were excluded, resulting in a relatively healthy cohort that does not fully represent the full spectrum of preterm-born children. As a result, while this limitation is particularly evident for preterm birth, the selective exclusion criteria in ABCD extend more broadly across the cohort, leading to potential underestimation of effects at the extremes of exposure and outcome severity and limiting generalizability to broader clinical and population distributions of neurodevelopmental conditions and risk profiles. Moreover, given the large sample size of the ABCD study, statistically significant findings may correspond to small effect sizes, and should therefore be interpreted with attention to their magnitude, consistency across studies, and potential practical relevance.

The ABCD assessment of preterm birth also has limitations: parents were asked “*Was the child born prematurely?*” and, if yes, “*About how many weeks premature?*”; some parents reported > 12 weeks, despite this being an exclusion criterion (Menu, Ji, et al., 2025). Analyses thus typically focus on preterm and early-term children, with these boundaries acknowledged. Additional study-specific exclusion criteria and participant self-selection can further reduce representativeness. Families who are harder to reach or face socioeconomic barriers may be more likely to attrit over time, and children with neurodivergent traits may move more in the scanner, reducing data quality or participation. Researchers often apply additional inclusion/exclusion criteria tailored to specific research questions, introducing further non-random selection. Stratified analyses, subgroup-sensitive approaches, and the use of sampling weights are therefore critical, as these methods help account for selection features and improve interpretability and generalizability (Gard et al., 2023). Finally, beyond study-level selection and representativeness issues, publication bias may also influence the literature synthesized here, as studies reporting null findings are less likely to be published or highlighted, potentially leading to an overrepresentation of positive associations and inflated effect consistency.

The breadth of the ABCD study’s outcomes is a strength and also introduces analytic complexity. Developmental trajectories may vary considerably by sociodemographic subgroup, and aggregate models may obscure meaningful heterogeneity. For example, associations between prenatal exposure and cortical thickness may vary by sex or socioeconomic status, but a single pooled model might fail to detect such interactions. Similarly, early-life environmental risk factors may interact with genetic predispositions, producing context-dependent effects that require nuanced modeling approaches. Sampling weights, stratified analyses, and interaction terms can help capture this heterogeneity, but careful interpretation remains essential.

Finally, ethical considerations are central: collection of sensitive maternal health and prenatal data requires strict privacy protections and transparency to maintain participant trust and ensure scientific integrity. Integration with complementary prospective birth cohorts (e.g., HBCD) and linkages to medical records could improve exposure precision and generalizability, while polygenic scores may help disentangle genetic confounding and test gene-environment interactions.

## 5 Unexplored and emerging opportunities

The 4-year follow-up presents a promising enhancement to the ABCD dataset. By pre-populating baseline responses and selectively updating key items, the follow-up allows not only for verification of prior reports but also for the inclusion of additional prenatal variables of interest, such as pre-eclampsia, eclampsia, and toxemia. This approach provides a unique opportunity to strengthen data reliability through targeted re-assessment, while simultaneously expanding the scope of prenatal exposures available for analysis. These methodological improvements set the stage for more detailed and nuanced investigations of early-life risk factors and developmental trajectories.

Building on this foundation, the ABCD study offers unique opportunities to advance understanding of prenatal and early-life determinants of neurodevelopment in ways that smaller or less comprehensive cohorts cannot. Its longitudinal design, with repeated follow-ups across childhood and adolescence, allows direct modeling of developmental trajectories rather than cross-sectional snapshots. In contrast, studies with only a few time points may still capture some longitudinal change but are often limited in their ability to reliably model complex, non-linear trajectories, potentially missing periods of rapid change or subtle developmental inflections. The integration of repeated neuroimaging, behavioral, and cognitive assessments with prenatal and early-life data enables person-centered approaches, such as latent class and trajectory-based models, to identify subgroups of children following distinct developmental pathways. Advanced analytic techniques, including machine learning, can leverage this multidimensional, temporally rich data to uncover complex, non-linear relationships and interactions among early exposures and later outcomes. Together, these features uniquely position ABCD to generate mechanistic insights, refine prediction of developmental risk and resilience, and inform targeted, individualized interventions, capabilities that traditional datasets are ill-equipped to provide.

A particularly promising emerging direction involves the use of postnatal neuroanatomical features as proxies for early prenatal developmental events. While birth weight has long served as a global retrospective indicator of fetal growth (Silventoinen et al., 2023), recent work has shifted toward identifying more specific markers of prenatal brain development. Beyond global cortical measures such as cortical thickness, volume, or surface area, increasing attention has been directed toward sulcal morphometry, including quantitative metrics (e.g. sulcal depth, width, length, sulcal surface area; Mangin et al., 2010) and qualitative features of cortical folding (Cachia et al., 2021). Recent work has demonstrated that these quantitative measures can be reliably extracted at scale using automated pipelines across large neuroimaging cohorts. For example, Pizzagalli and colleagues (2020) showed robust reliability of quantitative sulcal metrics across multiple datasets and demonstrated substantial heritability, as well as strong inter-hemispheric genetic correlations, of cortical folding features in cohorts comprising thousands of participants. Complementing these quantitative approaches, the sulcal, or sulcogyral, pattern is a macroscopic feature of cortical anatomy that reflects the topology of cortical folds (e.g., continuous vs. interrupted or broken sulci; presence vs. absence of specific folds) and their spatial organization (Cachia et al., 2021). Unlike quantitative features of the cortical sheet, such as thickness, surface area, or curvature/gyrification which continue to evolve over decades (Raznahan et al., 2011), qualitative sulcal patterns are largely determined before birth and remain remarkably stable across the lifespan (Cachia et al., 2016, Schwizer Ashkenazi et al., 2024). Recent empirical work further supports their sensitivity to intergenerational and prenatal influences: Mathan and colleagues (2024), for example, reported that anterior cingulate sulcal patterns are associated with parental socioeconomic status and global brain morphology, within a multivariate framework that also incorporates genetic liability, suggesting that prenatal neurodevelopmental processes may contribute to pathways linking socioeconomic conditions to brain development. Because of the high inter-individual variability of human sulcal morphology (Ono et al., 1990), traditional analyses have relied on expert visual inspection, an approach requiring extensive anatomical training and considerable time investment. Consequently, this methodology has never been applied to large-scale datasets such as ABCD. However, recent advances in deep learning have enabled fully automated, high-resolution recognition and classification of cortical folding patterns (Dufournet et al., 2025), offering a potentially scalable approach to studying sulcal morphology. These methods remain relatively new in neurodevelopmental research and will require further validation, standardization, and replication across independent cohorts before their broader applicability can be established. In this context, several promising research directions emerge for the ABCD cohort. Automated sulcal pattern analysis could, for instance, (i) identify early neuroanatomical correlates of prenatal adversity, (ii) serve as stable anatomical landmark for multimodal integration with structural, functional, and diffusion imaging data across time, and (iii) contribute to predictive models of later cognitive, emotional, or psychiatric outcomes. However, these applications should be viewed as preliminary and hypothesis-generating at this stage, pending further methodological validation.

Finally, the ongoing integration of ABCD findings with the HEALthy Brain and Child Development (HBCD; https://hbcdstudy.org/) study presents a compelling opportunity to extend early-life research across complementary cohorts. While ABCD follows children from late childhood into adolescence, HBCD focuses on the prenatal and the first decade of the child’s life, providing rich, prospective data on early exposures, including maternal health, environmental influences, and neurodevelopmental milestones. Linking insights from these studies can facilitate cross-cohort comparisons, enabling researchers to trace developmental trajectories from the earliest stages of life through later childhood and adolescence. This integration not only strengthens the study of prenatal and early-life determinants but also allows validation of patterns observed in ABCD, the identification of sensitive periods, and the generation of more comprehensive models of risk, resilience, and developmental heterogeneity (Dowling et al., 2024). Together, ABCD and HBCD offer an unprecedented framework for understanding the continuity and dynamics of neurodevelopment across the first two decades of life.

## 6 Implications for neuroscience and public health

Research leveraging the ABCD Study offers unparalleled opportunities to advance both neurodevelopmental science and public health. While the reliance on retrospective parental recall for prenatal exposures limits precision compared to prospective clinical cohorts, ABCD’s exceptional scale, longitudinal design, and multimodal approach enable robust examination of how early-life exposures are associated with brain-behavior trajectories and may relate to vulnerability or resilience across adolescence and early adulthood. By integrating neuroimaging, cognitive, behavioral, genetic, and environmental data, ABCD bridges biological and developmental perspectives, providing a powerful framework for understanding complex developmental mechanisms. Longitudinal evidence indicates that adult brain structure is strongly shaped by congenital and early-life factors, such as birth weight and polygenic risk, rather than within-person aging processes (Vidal-Pineiro et al., 2021), and that early-life influences on brain and cognition are often larger and more pervasive than later-life experiences (Walhovd et al., 2023), highlighting the critical importance of early development in shaping lifelong brain and cognitive health.

Beyond theory, ABCD also informs public health and policy. Evidence from the study has contributed to discussions around prenatal alcohol exposure (e.g., Lees et al., 2020) and shaped messaging on prenatal cannabis use (e.g., Paul et al., 2021). More broadly, these findings reinforce a key principle: prenatal development establishes foundational trajectories for lifelong cognitive, behavioral, and neural outcomes. This underscores a critical window for policy and intervention, while also leaving room for later experiences and supportive environments to modify these pathways, promoting resilience and healthy development. The integration of environmental datasets, including neighborhood disadvantage and opportunity indices (Cardenas-Iniguez et al., 2024), allows researchers to examine how socioeconomic and policy contexts may modulate associations between prenatal exposures and neurodevelopmental outcomes, for example by testing whether neighborhood disadvantage amplifies the impact of prenatal complications on cognitive and behavioral outcomes.

Moreover, ABCD findings provide insights relevant to intervention strategies. Programs like the Special Supplemental Nutrition Program for Women, Infants, and Children (WIC) demonstrate how targeting early-life risk can improve maternal and infant outcomes, including reductions in gestational diabetes, preterm birth, and neonatal ICU admissions (Venkatesh et al., 2024). Although ABCD is not an intervention study, its comprehensive dataset enables identification of modifiable targets and evaluation of broader population-level impacts, highlighting the translational potential of large-scale longitudinal research for informing policies that aim to improve developmental outcomes.

## 7 Conclusion

Taken together, evidence from the ABCD Study and related datasets highlights how prenatal and perinatal factors, such as maternal health, substance exposure, birthweight, and gestational age, are associated with long-term differences in brain development, cognition, and behavior. By integrating genetics, neuroimaging, environmental measures, and behavioral outcomes within a large, diverse, and longitudinal framework, ABCD provides a uniquely powerful platform for elucidating developmental pathways, refining neurodevelopmental theories, and tracing how early-life risks accumulate and interact over time. Future research should build on this foundation by linking ABCD with complementary datasets to clarify causal mechanisms, identify sensitive periods, and test how biological vulnerabilities are amplified or buffered by environmental contexts. In doing so, ABCD underscores the promise of longitudinal, multi-domain approaches for informing strategies that aim to optimize developmental outcomes from the very start of life.

## Funding

M.E.T. is supported by awards from the 10.13039/100000002National Institutes of Health, MH141129, MH126468, MH122447, DA055338 and ES032294.

## CRediT authorship contribution statement

**Moriah E. Thomason:** Writing – review & editing, Writing – original draft, Funding acquisition. **Iris Menu:** Writing – review & editing, Writing – original draft, Visualization, Methodology, Investigation, Conceptualization. **Arnaud Cachia:** Writing – review & editing, Writing – original draft.

## Declaration of Competing Interest

The authors declare that they have no known competing financial interests or personal relationships that could have appeared to influence the work reported in this paper.
